# Higher levels of tumor necrosis factor β are associated with frailty in socially vulnerable community-dwelling older adults

**DOI:** 10.1186/s12877-018-0961-6

**Published:** 2018-11-06

**Authors:** Carla M. C. Nascimento, Marisa S. Zazzetta, Grace A. O. Gomes, Fabiana S. Orlandi, Karina Gramani-Say, Fernando A. Vasilceac, Aline C. M. Gratão, Sofia C. I. Pavarini, Marcia R. Cominetti

**Affiliations:** Department of Gerontology, Federal University of São Carlos, Rod. Washington Luis, Km 235, Monjolinho, São Carlos, SP CEP 13565-905 Brazil

**Keywords:** Aging, Biomarkers, Older people, Frailty, Tumor necrosis factor β

## Abstract

**Background:**

The complex physiology underpinning the frailty syndrome is responsible for the absence of robust biomarkers that can be used for screening, diagnostic and/or prognostic purposes and has made clinical implementation difficult. Considering socially vulnerable populations, who have poor health status and increased morbidity and mortality, this scenario is even more complex. However, to the best of our knowledge, there are no studies available to investigate frailty biomarkers in socially vulnerable populations. Thus, the aim of this cross-sectional study was to identify potential blood-based biomarkers of frailty in a socially vulnerable population.

**Methods:**

A sample consisting of 347 community-dwelling older people living in a context of high social vulnerability was divided into non-frail (robust), pre-frail and frail groups, according to modified Fried frailty phenotype criteria. Blood samples were collected and analyzed for basic metabolic parameters and for inflammatory cytokines.

**Results:**

Levels of Interleukin-1α (IL-1α) and Tumor Necrosis Factor α (TNF-α) were significantly higher in pre-frail subjects, compared to non-frail ones. Tumor Necrosis Factor β (TNF-β) levels presented higher values in the frail compared to non-frail individuals. Interleukin-6 (IL-6) levels in pre-frail and frail subjects were significantly higher compared to the levels of non-frail subjects. Using an ordinal regression analysis, we observed that socially vulnerable older people at higher risk of developing frailty were subjects above 80 years old (OR: 2.5; 95% CI: 1.1–5.6) and who presented higher levels of TNF-β (≥0.81 pg/mL, OR: 2.53; 95% CI: 1.3–4.9).

**Conclusion:**

As vulnerable populations continue to age, it is imperative to have a greater understanding of the frailty condition, identifying novel potential blood-based biomarkers. The results presented here could help to implement preventive healthcare strategies by evaluating frailty and at the same time measuring a set of inflammatory biomarkers, paying special attention to TNF-β plasmatic levels.

**Electronic supplementary material:**

The online version of this article (10.1186/s12877-018-0961-6) contains supplementary material, which is available to authorized users.

## Background

Frailty is an age-related state characterized as a syndrome in which individuals may become more vulnerable to adverse health outcomes with a higher risk for mortality when exposed to stressors [1–3]. Social vulnerability is defined as the degree to which a person’s overall social situation leaves them susceptible to health problems, which include physical, mental, psychological and functional problems. Social vulnerability is particularly important for older adults as a condition related to cultural, social and economic aspects that may influence the access to goods and services. This is an important indicator to explain the high exposure of this population to the development of pathological and degenerative conditions as it increases the frailty rate [4–8].

On the other hand, knowledge regarding the cellular and molecular mechanisms related to this syndrome is limited, especially in vulnerable populations. Changes related to the immune-endocrine axis are extensively described during aging and are frequently associated to increases in morbidity and mortality [9]. These changes are characterized by a low-grade, controlled, asymptomatic, systemic, and chronic progressive increase in the pro-inflammatory status, also called *inflammaging* [10]. This pro-inflammatory state generated by age, sex, lifestyle, socioeconomic background, comorbidities and affective, cognitive or sensory impairments has been postulated as a potential driver of frailty pathogenesis, producing even higher levels of systemic inflammatory markers [11, 12]. Associations between frailty and endocrine and immune changes have been demonstrated, suggesting that alterations in these systems may accelerate the development of age-related diseases and consequently, frailty. [13, 14]. Considering that frailty is characterized by the loss of resilience, poor social conditions may reflect chronic stressors, which increase risk of disturbances in metabolic and immunological parameters, leading to a higher risk of developing frailty [15–17].

Previous studies have pointed out several candidates for blood-based biomarkers for frailty [18]. Those include inflammatory molecules, such as C-reactive protein (CRP) [19, 20], Interleukin-6 (IL-6) [19–22] and Tumor Necrosis Factor-α (TNF- α) [19, 21, 23], clinical parameters such as hemoglobin [19, 24] and serum albumin [25], hormones, such as dehydroepiandrosterone sulfate (DHEA-S) [26], testosterone [27], Insulin-like Growth Factor-1 (IGF-1) [28] and vitamin D [29], among others. To the best of our knowledge, however, no study has described the differences of clinical parameters and pro-inflammatory cytokines so far among different frailty statuses of older adults living in socially vulnerable populations.

As a result, we performed a cross-sectional study to investigate potential blood-based biomarkers for frailty under a social vulnerability context, considering demographic, psychological and clinical characteristics. The results of this study could contribute to a greater understanding of frailty syndrome in socially vulnerable populations, as well as to the biology of frailty with regards to the panel of expressed inflammatory cytokines. Moreover, identifying potential blood-based biomarkers will enable the development of interventions and specific policies for this public.

## Methods

### Sample

A total of 852 older adults fulfilled the eligibly criteria and were registered in the database. Based on this, the adequate sample size was calculated according to the multinomial statistics analysis. Thus, 347 representative participants were stratified according to sex and age sample. This sample size provided a power of 95%, considering a medium effect size (50%; *p* = 0.50). Participants who accepted to take part in the study and signed the consent term were asked to collect blood samples. Immediately after drawing blood, the clinical assessment for frailty status was scheduled.

### Study protocol

The study protocol (860.653/2014) and informed consent form received ethics approval from the Federal University of São Carlos Ethics Committee on Human Experimentation. Written informed consent concerning the conduct of the survey was obtained from each participant, and complete anonymity of participants was assured as described earlier [30].

Brazil has a Unified Health System (*Sistema Unico de Saúde - SUS*), and it is estimated that more than 75% of the Brazilian population rely exclusively on it for their health care. The Family Health Program is a part of the unified health system created to provide mainly primary care health [31]. Since we consider that these units are located mainly in vulnerable areas, most people living in these zones are users and are registered on the *SUS* database. All people that use the health care service must be registered and accompanied by a team of health professionals. For this study, older adults (60 years and over) were selected, who were registered in the *SUS* database in a region with a high rate of social vulnerability and poverty in São Carlos, São Paulo, Brazil, called “*Cidade Aracy*”, which is a region with a high rate of poverty that unifies five public clinical practice units. Social vulnerability was determined using the Paulista Social Vulnerability Index (PSVI) that measures social exclusion in different cities in São Paulo state, Brazil [32]. SVI allows the classification of a determined population into seven groups: 1 - extremely low; 2 - very low; 3 - low; 4 - medium; 5 - high; 6 - very high vulnerability and 7 - rural areas of high vulnerability (Additional file 1). Based on these criteria, the study population was classified as level 5 of vulnerability, which represents populations living in urban areas with high vulnerability. This instrument was used with the unique intention to determine the social vulnerability of the studied population, not for comparison purposes.

Subjects were classified according to diagnostic criteria based on the classification proposed by Fried and co-workers [1] and validated in Brazil by Nunes and colleagues [33], as described in the study protocol. According to these criteria, the subjects were classified into three groups: frail (scores 3–5), pre-frail (scores 1, 2), and non-frail (score 0). For the diagnosis of frailty, five items were considered: 1) non intentional weight loss was evaluated by means of self-report considering losses of more than 4.5 kg during the last year; 2) fatigue was assessed considering the answers given to two items from the Center of Epidemiological Study Center Scale (CES-D): “*Have you felt that you had to make an effort to do your customary tasks*?” and “*Were you unable to proceed when doing your things*?” [34]; 3) Handgrip strength was measured using a manual dynamometer and was considered the measure of the highest force possible produced for the dominant upper limb in three attempts. These values were adjusted considering gender and body mass index (BMI) for the final criteria; 4) physical inactivity was evaluated by applying the International Physical Activity Questionnaire (IPAQ) [35], which assesses the amount of physical activity performed by the individual and the estimated caloric expenditure. Individuals with a caloric expenditure in the first quintile were considered positive for this item; 5) low-gait speed was measured using a 4.6-m-long circuit and the time spent to go through the circuit. The individuals in the first quintile, after adjusting their respective heights and genders, were considered positive for this item.

Sociodemographic and clinical data were also assessed: age, sex, education, ethnicity, cognitive function measured by the Mini Mental State Examination [36], depressive symptoms measured by the Geriatric Depression Scale, GDS-15, Brazilian version [37], leisure-time of physical activity measured by the self-report specific part of the International Physical Activity Questionnaire [35], medications, Body Mass Index (BMI), marital status and per capita income. In an effort to avoid potential sources of bias, all the interviews were conducted by trained gerontologists and data was included using the software Epidata® software.

### Analysis of biomarkers

Fasting blood samples were collected on a visit closest to the frailty assessment. Samples were collected using EDTA tubes, mixed by inversion and centrifuged (3000 rpm, 5 min) to separate plasma. Plasma samples were stored at − 80 °C until they were analyzed. One plasma aliquot was used to perform measures of glucose, insulin, total cholesterol, triglycerides, urea, creatinine, dehydroepiandrosterone (DHEA), dehydroepiandrosterone sulfate (S-DHEA), human growth hormone (GH), insulin-like growth factor 1 (IGF-1) and glycated hemoglobin in a specialized clinical laboratory.

Another aliquot was used to measure the level of inflammatory cytokines. For that, 25 μL of samples were added to each well of a 96-well plate provided with the kit (HCYTOMAG, MILLIPLEX® MAP Kit EMD Millipore Corp., MA, USA). Plates were incubated under agitation on a shaker for 2 h at room temperature (20–25 °C). Next, after washing according to the instructions provided in the kit, 25 μL of premixed beads containing the primary antibodies to detect the specific cytokines evaluated (Interleukin-1α; Interleukin-1β; Interleukin-6; Tumor Necrosis Factor α and Tumor Necrosis Factor β) were inserted in each well. The plate was sealed and incubated for 2 h at room temperature (20–25 °C). Afterwards, the plates were washed twice with the washing solution provided and 25 μL of streptavidin-phycoerythrin solution was added to each well and plates were incubated with agitation on a shaker for 30 min at room temperature. A standard curve was prepared with the reagents provided in the kit and luminescence readings were performed on a plate reader (Luminex xMAP®).

### Statistical analysis

To describe the profile samples for all variables, data were presented in frequency tables for categorical data and descriptive statistics were applied for numerical data with means and standard deviation. In order to compare categorical data, the chi-square and the Fisher exact tests were used. When comparisons involved three levels of frailty (non-frail, pre-frail and frail individuals), the *Kruskal-Wallis* test was used due to the non-normal distribution of the data. To compare numerical variables according to frailty status adjusted for sex and age, an analysis of covariance (ANCOVA) was applied. Univariate analysis was performed to assess the association between factors and outcome measures. Variables considered with a significance of at least 20% (p < =0.20) in the univariate analysis were all included in the multivariable ordinal logistic regression. In this model, variables were considered significant at *p* ≤ 0.05. All statistical analyses were performed using the SAS System for Windows (Statistical Analysis System), version 9.2 (SAS Institute Inc., 2002–2008, Cary, NC, USA).

## Results

The mean age of the study cohort was 70.1 ± 7.7 years, mostly female (56.2%) with a low education level (3.8 ± 2.3 years) (data not shown). In this sample, 34 subjects (9.8%) were non-frail, 197 (56.8%) were classified as pre-frail and 116 (33.4%) subjects fulfilled the criteria for physical frailty. Considering age, as expected, frail individuals were significantly older than their non-frail counterparts (Table 1).

**Table 1:** Sociodemographic characteristics and clinical parameters of participants distributed according to the Fried frailty scale, *n* = 347

	Non-frail	Pre-frail	Frail	*p-value*
	*n* = 34 (9.8%)	*n* = 197 (56.8%)	*n* = 116 (33.4%)	
Age (%)				**0.003**
60–69 years	52.9	59.4^*^	43.1	
70–79 years	47.1^*^	28.4	37.1	
> 80 years	0.0	12.2	19.8^*^	
Sex (Men/women, %)	20.6/79.4^*^	43.1^*^/56.9	51.7^*^/48.3	**0.005**
Schooling (%)				0.161
0 years	0.0	1.5	4.3	
1–4 years	76.9	72.8	82.6	
> 5 years	23.1	25.7	13.1	
Ethnicity (White/non-white, %)	44.1/55.9	39.5/50.5	43.9/56.1	0.700
Global Cognitive State-MMSE (%)				0.132
Without cognitive impairment	58.8	60.7	49.1	
With cognitive impairment	41.2	39.3	50.9	
Depressive Symptoms-GSD (%)	11.8	31.1^*^	40.5^*^	**0.006**
Physically Active Enough-IPAQ score (%)	73.5	61.9	29.3^*^	**< 0.001**
Medications (%)				0.388
0–5 medications	75.0	67.8	62.3	
> 6 medications	25.0	32.2	37.7	
Body Mass Index	29.5 ± 6.5	28.6 ± 5.9	28.2 ± 6.1	0.906
Marital status (With/without partner, %)	64.7/35.3	59.7/40.3	52.6/47.4	0.325
Per capita income	280.4 ± 151.3	239.2 ± 127.7	220.3 ± 126.5	0.169

*Notes:* Data are expressed as mean ± SD or percentage of total sample; Body Mass Index was calculated as weight in kilograms divided by height in square meters. GDS: Geriatric Depression Scale; MMSE: Mini-Mental State Examination. Per capita incomes are in US dollars, according to the quotation made on October/2017. Chi-square test (χw^2^) was used for categorical variables and Kruskal-Wallis test for numerical variables. *Values with statistical significance (*p* < 0.05)

All other bold entries represent the *p* values of significant results

Results showed significant differences between groups concerning the sex, indicating that women were more frequent in the non-frail group, while men were most frequent in pre-frail and frail categories. Regarding age, as expected, older people (> 80 years) were frailer compared to those in the age groups of 60–69 and 70–79 years. The Geriatric Depression Scale (GDS) indicated that prevalence of depressive symptoms was also higher in pre-frail and frail, compared to non-frail individuals. Frail individuals also presented insufficient levels of physical activity evaluated by the International Physical Activity Questionnaire (IPAQ) (Table 1), which is consistent to the frailty condition.

Considering the results of metabolic parameters and biomarker evaluation, we observed that urea and TNF-β were significantly higher in frail participants compared to non-frail individuals. Levels of creatinine and IL-6 were higher among pre-frail and frail individuals, compared to non-frail ones. Cytokines IL-1α and TNF-α levels were higher in pre-frail, compared to non-frail, but no statistically significant differences for these cytokines were found comparing non-frail with frail individuals (Table 2).

**Table 2:** Peripheral biomarkers of participants distributed according to the Fried frailty scale, *n* = 347

	Non-frail *n* = 34 (11.2%)	Pre-frail *n* = 197 (58.2%)	Frail *n* = 116 (30.6%)	*p-value*
Glucose (mg/dL)	95.5 ± 27.5	98.7 ± 44.5	106.4 ± 52.2	0.640
Glycated hemoglobin (%)	5.8 ± 1.7	5.7 ± 2.4	5.7 ± 2.8	0.874
Insulin (μmol/L)	8.4 ± 5.5	8.6 ± 7.8	8.7 ± 8.1	0.663
Total cholesterol (mg/dL)	200.2 ± 40.4	195.2 ± 43.1	193.3 ± 45.7	0.435
HDL cholesterol (mg/dL)	49.4 ± 11.2	48.3 ± 13.5	47.2 ± 11.6	0.497
LDL cholesterol (mg/dL)	119.8 ± 37.8	119.6 ± 34.2	115.2 ± 34.2	0.441
VLDL cholesterol (mg/dL)	32.3 ± 23.0	28.4 ± 13.6	30.2 ± 16.3	0.818
Triglycerides (mg/dL)	146.2 ± 61.2	148.5 ± 82.7	151.4 ± 89.7	0.939
Urea (mg/dL)	34.5 ± 10.1	35.5 ± 11.6	40.1 ± 14.9	0.013* ^a^
Creatinine (mg/dL)	0.8 ± 0.2	0.96 ± 0.3	1.1 ± 0.5	0.004* ^a,b^
DHEA (ng/mL)	4.1 ± 4.5	4.4 ± 4.3	4.3 ± 4.5	0.407
S-DHEA (μg/dL)	55.8 ± 40.3	67.2 ± 48.4	67.2 ± 53.3	0.423
IGF-1 (ng/mL)	113.2 ± 42.4	119.7 ± 47.5	117.2 ± 43.2	0.776
Hydroxyvitamin D (ng/mL)	22.1 ± 5.6	23.2 ± 8.6	22.2 ± 7.8	0.693
Inflammatory markers (pg/mL)				
Interleukin-1α	3.0 ± 5.2	6.8 ± 12.5	9.2 ± 21.8	0.027* ^b^
Interleukin-1β	1.9 ± 2.8	3.1 ± 4.2	3.4 ± 4.4	0.056
Interleukin-6	1.9 ± 1.6	2.1 ± 4.2	4.2 ± 7.6	0.012* ^a,b^
Interleukin-10	1.3 ± 2.0	1.7 ± 3.2	3.6 ± 12.4	0.571
Tumor Necrosis Factor-α	2.0 ± 2.0	3.6 ± 5.8	4.7 ± 10.2	0.035* ^b^
Tumor Necrosis Factor-β	0.5 ± 0.5	3.0 ± 11.5	3.0 ± 10.1	0.033* ^a^

*Notes:* DHEA: Dehydroepiandrosterone; S-DHEA: Dehydroepiandrosterone sulfate; GH: Growth Hormone; IGF-1: Insulin-like growth factor 1. Kruskal-Wallis test. ^*^Values with statistical significance (*p* < 0.05). ^a^Non-frail vs frail; ^b^non-frail vs pre-frail

All other bold entries represent the *p* values of significant results

We performed a univariate regression analysis to track the main variables that seem to contribute to the frailty phenotype considering the three groups (frail vs pre-frail vs non-frail) evaluated. Variables were considered to be part of the ordinal model when they presented a statistical significance above 20% (*p* < 0.20). Thus, we considered the following variables for our model: sex, age, HDL and VLDL cholesterols, urea, creatinine, triglycerides IL-1β, IL-6 and TNF-β (Table 3).

**Table 3:** Results of the univariate ordinal logistic regression analysis among groups (frail vs pre-frail vs non-frail)

		Univariate Regression		
Variable	Category	O.R.	95% CI	*p-*value
Sex	Women (ref.) Men	1.00 1.85	--- 1.21–2.82	--- 0.004*
Age (years)	60–69 years (ref.)	1.00	–	–
	70–79 years	1.29	0.81–2.05	0.278
	≥80 years	2.65	1.41–5.00	0.003*
Glucose (mg/dL) (tertile)	≤83 (ref.)	1.00	–	–
	84–96	0.99	0.59–1.67	0.979
	≥97	1.06	0.64–1.77	0.816
HDL cholesterol (mg/dL) (tertile)	≥51 (ref.)	1.00	–	–
	42–50	1.18	0.70–1.97	0.538
	≤41	1.42	0.85–2.40	0.184*
LDL cholesterol (mg/dL) (tertile)	≤99 (ref.)	1.00	–	–
	100–129	1.18	0.70–1.98	0.531
	≥130	0.83	0.49–1.41	0.493
VLDL cholesterol (mg/dL) (tertile)	≤20 (ref.)	1.00	–	–
	21–31	0.59	0.35–1.01	0.051*
	≥32	1.00	0.59–1.69	1.000
Urea (mg/dL) (tertile)	≤30 (ref.)	1.00	–	–
	31–39	1.21	0.71–2.08	0.480
	≥40	2.01	1.19–3.41	0.010*
Creatinine (mg/dL) (tertile)	≤0.7 (ref.)	1.00	–	–
	0.8–1.0	0.85	0.48–1.50	0.569
	≥1.1	1.96	1.05–3.66	0.034*
DHEA (ng/mL) (tertile)	≤2.31 (ref.)	1.00	–	–
	2.32–4.28	0.90	0.54–1.52	0.699
	≥4.29	0.98	0.58–1.63	0.922
Hydroxyvitamin D (ng/mL) (tertile)	≥25 (ref.)	1.00	–	–
	19–24	0.96	0.58–1.59	0.880
	≤18	1.10	0.66–1.84	0.720
Insulin (μmol/L) (tertile)	≥9 (ref.)	1.00	–	–
	5–8	1.31	0.79–2.16	0.298
	≤4	0.93	0.55–1.55	0.769
Triglycerides (mg/dL) (em tertile)	≤103 (ref.)	1.00	–	–
	104–158	0.53	0.32–0.90	0.019*
	≥159	0.88	0.53–1.47	0.627
Total cholesterol (mg/dL) (tertile)	≤174 (ref.)	1.00	–	–
	175–211	1.16	0.69–1.94	0.577
	≥212	0.72	0.43–1.21	0.211
S-DHEA (μg/dL) (tertile)	≤36 (ref.)	1.00	–	–
	37–73	1.17	0.69–1.97	0.556
	≥74	1.37	0.81–2.30	0.243
IGF-1 (ng/mL) (tertile)	≤91.5 (ref.)	1.00	–	–
	91.6–134.3 ≥134.4	0.98 1.14	0.58–1.64 0.68–1.91	0.932 0.621
Glycated hemoglobin (%) (tertile)	≤5.5 (ref.)	1.00	–	–
	5.6–6.3	0.70	0.41–1.17	0.173
	≥6.4	0.90	0.54–1.51	0.694
IL-10 (pg/dL) (tertile)	≤0.39 (ref.)	1.00	–	–
	0.40–1.05	1.11	0.63–1.96	0.720
	≥1.06	1.31	0.74–2.32	0.362
IL-1α (pg/dL) (tertile)	≤0.60 (ref.)	1.00	–	–
	0.70–3.35	1.30	0.74–2.28	0.355
	≥3.36	1.22	0.69–2.13	0.498
IL-1β (tertile)	≤0.86 (ref.)	1.00	–	–
	0.87–2.30	1.21	0.69–2.12	0.507
	≥2.31	1.47	0.83–2.59	0.183*
IL-6 (tertile)	≤1.50 (ref.)	1.00	–	–
	1.51–2.60	1.62	0.96–2.72	0.071*
	≥2.61	1.67	0.99–2.81	0.052*
TNF-α (tertile)	≤0.96 (ref.)	1.00	–	–
	0.97–2.89	0.92	0.53–1.61	0.776
	≥2.90	0.99	0.57–1.76	0.993
TNF-β (tertile)	≤0.29 (ref.)	1.00	–	–
	0.30–0.80	1.24	0.70–2.20	0.459
	≥0.81	2.39	1.33–4.28	0.003*

Variables with P < 0.20 were entered in the multivariable logistic regression model, *n* = 347. *Notes:* OR: Odds ratio shows the increase in odds for frailty. 95% CI = 95% of confidence interval for odds ratio; ref. = category chosen as reference. *Values with statistical significance (*p* < 0.2)

All other bold entries represent the *p* values of significant results

Given these results, an ordinal regression model was conducted in order to predict which variables were significantly associated with frailty. Results showed that age and TNF-β levels were significantly associated with frail phenotype. Older people with increased risk to becoming frail were those older than 80 years (OR: 2.5; 95% CI: 1.09–5.61) and who presented higher levels of TNF-β (OR: 2.5; 95% CI: 1.30–4.9) (Table 4).

**Table 4:** Results of multivariable ordinal logistic regression for frailty (*n* = 257)

Variable	Category	O.R.	95% CI	*p*-value
Sex	Female (ref.)	1.00	–	–
	Male	1.61	0.89–2.92	0.116
Age	60–69 years (ref.)	1.00	–	–
	70–79 years	1.26	0.72–2.21	0.422
	≥80 years	2.47	1.09–5.61	0.031*
HDL (mg/dL, tertile)	≥51 (ref.)	1.00	–	–
	42–50	1.02	0.55–1.89	0.942
	≤41	1.03	0.53–2.02	0.931
VLDL (mg/dL, tertile)	≤20 (ref.)	1.00	–	–
	21–31	1.49	0.21–10.51	0.687
	≥32	1.38	0.09–21.22	0.818
Urea (mg/dL, tertile)	≤30 (ref.)	1.00	–	–
	31–39	1.28	0.66–2.46	0.469
	≥40	1.73	0.86–3.45	0.123
Creatinine (mg/dL, tertile)	≤0.7 (ref.)	1.00	–	–
	0.8–1.0	0.67	0.33–1.34	0.256
	≥1.1	1.21	0.51–2.91	0.668
Triglycerides (mg/dL, tertile)	≤103 (ref.)	1.00	–	–
	104–158	0.39	0.06–2.78	0.350
	≥159	0.72	0.05–10.92	0.810
IL-1β (pg/mL, tertile)	≤0.86 (ref.)	1.00	–	–
	0.87–2.30	1.05	0.54–2.05	0.890
	≥2.31	0.88	0.44–1.74	0.707
IL-6 (pg/mL, tertile)	≤1.50 (ref.)	1.00	–	–
	1.51–2.60	1.80	0.92–3.54	0.088
	≥2.61	1.84	0.93–3.64	0.078
TNF-β (pg/mL, tertile)	≤0.29 (ref.)	1.00	–	–
	0.30–0.80	1.14	0.59–2.18	0.698
	≥0.81	2.53	1.30–4.90	0.006*

*Notes:* OR (*Odds Ratio*) = risk of frailty. 95% CI = 95% Confidence interval for odds ratio. *Values with statistical significance (*p* < 0.05)

Score Test for the Proportional Odds Assumption: X2 = 17.59; DF = 19; *P* = 0.550; *n* = 29 non-frail, *n* = 143 pre-frail and *n* = 85 frail

All other bold entries represent the *p* values of significant results

## Discussion

Frailty is a geriatric syndrome associated with disability and mortality outcomes in older people. In a recent multicenter, a population-based cross-sectional study showed a prevalence around 38% of frailty in Brazilian community-dwelling older people [38]. We identified in our sample, in which all individuals were living in a high social vulnerability context, that 33.4% of aged subjects investigated fulfilled criteria for physical frailty. A possible explanation for these differences may be related to the fact that in our study, most of the participants were in the 60 to 69 year old age group (53.3%; data not shown) and few participants were above 80 years old (13.5%; data not shown), while in a study carried out by Pereira et al. [38] the age group ranging from 60 to 69 years old represented only 36.4% of the sample. More importantly, in the study by Pereira et al. [38], the authors did not evaluate frailty using Fried’s score, but instead used the frailty index (FI), which probably contributed to the difference found in both studies, regarding frailty prevalence. Accordingly, Collard et al. [39], in a systematic review of 21 studies (*n* = 61,500), described that the prevalence of frailty in a community-dwelling population has a high variability, ranging from 4 to 59.1%. Moreover, in another study, Moreira and Lourenço [40] found a prevalence of 9.1% of frailty when a different scale was applied to track this phenotype, while Sousa et al. [41] found a prevalence of 17.1% for Brazilian older people using Fried’s phenotype scale. These variations may be highlighted by the different operationalization of frailty status used in the studies, resulting in widely different frailty prevalences.

The frail condition in our sample was also more frequent in men, which is different from previously published epidemiological data that showed higher rates of frailty in women [42, 43]. It has been demonstrated that men frequently have more acute illnesses, while women have more comorbidities and disabilities [44, 45]. This may consequently lead to men becoming more vulnerable to frailty in advanced ages. Additionally, it is important to consider that Brazilian population social inequalities in health behavior are very relevant to explain the particularity of some results found for our sample. It has already been demonstrated that in low-income, less schooled people and those without private health insurance, Brazilian men were found to have more risk behavior, including smoking, sedentary lifestyles and bad quality of food, with a low intake of fruit and vegetables [46]. Furthermore, another investigation found that around 1.1% among men and 0.3% among women in this social condition have never visited a physician and 35% and 52% of men and women respectively, had not visited a physician in the last year [47]. These findings help to elucidate the underutilization of health care services among individuals of the lowest economic class, but especially among men, which may characterize them as a more vulnerable group for frailty. On the other hand, our data is in accordance with other research, indicating that frailty increases with age and low levels of physical activity [39].

The aging process frequently results in a decline of the immune function, known as immunosenescense, which was related to the role of inflammation in frailty [48]. These alterations are characterized by around 2-fold increased levels of cytokine production when compared to young individuals’ levels. Increases in adipose tissue, low levels of physical activity and the senescent cellular process itself were suggested as mainly factors to drive this phenomenon that results in a low-grade chronic inflammatory state [49]. However, it is difficult to establish the relationship between inflammation and frailty, considering that both conditions linearly increase with advancing age, thus avoiding a clear answer to determine whether inflammation is a cause or a consequence of the frailty phenotype. Nevertheless, in a recent meta-analysis involving 32 cross-sectional studies and 23,910 older people, it was demonstrated that frail and pre-frail conditions are related to increased levels of serum cytokines, when compared to the levels of non-frail participants [50]. Our results corroborate the literature, demonstrating that an increased pro-inflammatory state is also present in frail older adults living in a context of social vulnerability.

Using an ordinal regression model, we found that TNF-β was significantly associated to the frail phenotype. Abnormalities in TNF-α or TNF-β expression have been implicated in several diseases [51–53]. A TNF-β signaling pathway seems to be involved in the support of efficient immune responses against pathogens, which is a key point in the immunosenescense [54]. An important link between the lack of adaptability of immunological function during aging and increased risk for frailty in the vulnerable oldest old individuals was found in our study and may indicate that imbalances in immune functions seem to trigger physical frailty in this population. Similarly, in a systematic and meta-analysis review, Soysal and co-workers [50] demonstrated that three large prospective studies failed to find any association between higher inflammatory levels and frailty. The authors attribute this result to the paucity of data, to an over-adjustment of the analyses and/or to the fact that frail people were prone to diseases that might have increased the levels of inflammatory cytokines during the follow-up period and the lack of an adjustment for inherent changes to these markers in their analyses.

As an important limitation of this work, we can point out that this is a cross-sectional study, which does not follow individuals over time. A follow-up study using the same approaches to investigate frailty in this population is currently underway in our group. On the other hand, the statistical power of our sample size allows the expansion of our findings to other already recognized socially vulnerable populations. Notwithstanding, the knowledge of the characteristics related to socioeconomic and biological conditions configures an important tool to raise preventive strategies to decrease frailty risk in the most vulnerable populations.

## Conclusions

Taken together, the results presented here could help to implement preventive healthcare strategies by evaluating frailty and at the same time measuring a set of inflammatory biomarkers, paying special attention to TNF-β plasmatic levels. This could enable health teams to plan and improve care actions for this population, especially in the community and in primary health services.

## Additional file

Additional file 1: Description of the Paulista Social Vulnerability Index. (DOCX 19 kb)

## Abbreviations

BMI: Body Mass Index
DHEA: Dehydroepiandrosterone
DHEA-S: Dehydroepiandrosterone sulfate
GDS: Geriatric Depression Scale
GH: Human Growth Hormone
IGF-1: Insulin-like Growth Factor-1
IL-1α: Interleukin-1α
IL-1β: Interleukin-1β
IL-6: Interleukin-6
IPAQ: International Physical Activity Questionnaire
SVI: Social Vulnerability Index
TNFα: Tumor Necrosis Factor α
TNFβ: Tumor Necrosis Factor β
