# Supplementary material for: Higher levels of tumor necrosis factor β are associated with frailty in socially vulnerable community-dwelling older adults
Source: BMC Geriatr. 2018 Nov 6;18:268. doi: 10.1186/s12877-018-0961-6 (PMC6219095; doi:10.1186/s12877-018-0961-6)
Supplement: Supplementary file 1 — Description of the Paulista Social Vulnerability Index. (DOCX 19 kb) [file 12877_2018_961_MOESM1_ESM.docx]

**Higher levels of Tumor Necrosis Factor β are associated with frailty in socially vulnerable community-dwelling older adults**

Carla M. C. Nascimento (carlamcnascimento@gmail.com), Marisa S. Zazzetta, (marisam@ufscar.br), Grace A. O. Gomes (grace@ufscar.br), Fabiana S. Orlandi (forlandi@ufscar.br), Karina Gramani-Say, (gramanisay@ufscar.br), Fernando A. Vasilceac (fervasilceac@ufscar.br), Aline C. M. Gratão (alinegratao@ufscar.br), Sofia C. I. Pavarini (sofia@ufscar.br), Marcia R. Cominetti ([mcominetti@ufscar.br](mailto:mcominetti@ufscar.br))

**Supplementary material**

**Paulista Social Vulnerability Index^[[1]](#footnote-1)^**

**Group 1 (very low vulnerability):** In the space occupied by these sectors, the average nominal income of households was R$ 8,459^[[2]](#footnote-2)^ and in 1.4% of them the income did not exceed minimum wage per capita. With regard to demographic indicators, the average age of heads of households was 48 and those under 30 represented 12.6%. Among the female heads of households, 14.0% were under 30 years old, and the share of children from 0 to 5 years was equivalent to 5.9% of the total population of this group.

**Group 2 (very low vulnerability):** In the space occupied by these sectors, the average nominal income of households was R$ 2,964 and in 8.1% of them the income did not exceed minimum wage per capita. With regard to demographic indicators, the average age of heads of households was 50 and those under 30 represented 9.6%. Among the female heads of households, 8.8% were under 30 years old, and the share of children from 0 to 5 years old equaled 6.3% of the total population of this group.

**Group 3 (low vulnerability):** In the space occupied by these census tracts, the average nominal household income was R$ 2,133 and in 14.0% of them the income did not exceed half a minimum wage per capita. With regard to demographic indicators, the average age of the heads of households was 42 years and those who were less than 30 years represented 21.0%. Among female heads of households, 22.4% were aged 30 or over, and the share of children from 0 to 5 years old equaled 9.0% of the total population of this group.

**Group 4 (average vulnerability - urban sectors):** In the space occupied by these census sectors, the average nominal household income was R$ 1,627 and in 22.0% of them the income did not exceed the average minimum wage per capita. With regard to demographic indicators, the average age of heads of households was 47 years and those under 30 represented 12.1%. Among female heads of households, 9.7% were up to 30 years old, and the share of children aged 0 to 5 years was equivalent to 8.4% of the total population of this group.

**Group 5 (high vulnerability - urban sectors):** In the space occupied by these census sectors, the average nominal household income was R$ 1,401 and in 28.7% of them the income did not exceed an average minimum wage per capita. With regard to demographic indicators, the average age of heads of households was 42 years and those under 30 represented 20.3%. Among female heads of households, 20.6% were under 30 years old, and the share of children aged 0 to 5 years was equivalent to 10.5% of the total population of this group.

**Group 6 (very high vulnerability - subnormal clusters):** In the space occupied by these census tracts, the average nominal household income was R$ 1,201 and 34.9% of the income exceeded one-half minimum wage per capita. With regard to demographic indicators, the average age of those responsible by households was 40 years and those under 30 represented 22.6%. Among the women heads, 22.7% were under 30 years old and the proportion of children aged 0 to 5 years was equivalent to 11.3% of the total group.

**Group 7 (high vulnerability - rural sectors):** In the space occupied by these sectors, the average nominal household income was R$ 1,054 and in 42.5% of them the income did not exceed half of the minimum wage per capita. With regard to demographic indicators, the average age of heads of households was 48 and those under 30 represented 13.1%. Among the female heads of household, 13.7% were under 30 years old, and the share of children from 0 to 5 years was equivalent to 9.2% of the total population of this group.

1. Extracted from the web page of the Legislative Assembly of the State of São Paulo at: <http://indices-ilp.al.sp.gov.br/view/index.php?prodCod=2>, on September 21^st^, 2018. [↑](#footnote-ref-1)
2. R$ 1.00 is equivalent to U$ 0.24, consulted on September 21^st^, 2018. [↑](#footnote-ref-2)
